# Spontaneous pneumothorax with pulmonary Langerhans cell histiocytosis (PLCH) in an adult heavy cigarette smoker—A case report

**DOI:** 10.1002/rcr2.939

**Published:** 2022-03-27

**Authors:** Chiao‐Yun Tsai, Hsu‐Chih Huang, Zhen‐Dong Xu, Jiun‐Yi Hsia, Chih‐Yi Chen

**Affiliations:** ^1^ Division of Thoracic Surgery, Department of Surgery Chung Shan Medical University Hospital Taichung Taiwan, Republic of China; ^2^ Institute of Medicine Chung Shan Medical University Taichung Taiwan, Republic of China; ^3^ Department of Pathology Chung Shan Medical University Hospital Taichung Taiwan, Republic of China; ^4^ School of Medicine Chung Shan Medical University Taichung Taiwan, Republic of China

**Keywords:** CD1a‐positive histiocyte‐like cells, ground‐glass nodules, heavy cigarette smoker, high‐resolution computed tomography (HRCT), lung biopsy, lung‐infiltrated parenchyma cysts, minimal invasive thoracoscopic surgery, pleurodesis, pulmonary histiocytosis X, pulmonary Langerhans cell histiocytosis (PLCH), smoking cessation, spontaneous pneumothorax

## Abstract

Pulmonary Langerhans cell histiocytosis is a rare disease caused by the proliferation of CD1a‐positive histiocyte‐like cells infiltrating the lung's interstitial layer. Most cases affect young to middle‐aged persons, especially adult heavy cigarette smokers. A 49‐year‐old male heavy smoker (40 pack‐year), with non‐productive cough, dyspnoea and desaturation, presented with a right‐sided pneumothorax on chest x‐ray with total atelectasis. Chest computed tomography (CT) revealed bilateral multiple thick‐walled infiltrated cysts and multiple ground‐glass nodules throughout the entire lung. Surgery with minimal invasive thoracoscopic lung biopsy and pleurodesis was performed. Pathology showed histiocyte‐like cells aggregates in the pulmonary parenchyma. Immunohistochemical stain demonstrated CD1a(+), S100(+) and CD68(+). After 3 months of smoking cessation, clear improvement was evidenced with a chest CT showing bilateral multiple thin‐walled rounded cysts and multiple ground‐glass nodules that are smaller in size and decreased in numbers. Early minimal invasive thoracoscopic lung biopsy and pleurodesis can also be a choice if the development of secondary spontaneous pneumothorax occurs.

## Graphical abstract

Primary therapy of pulmonary Langerhans cells histiocytosis is smoking cessation. Early surgical intervention by minimal invasive thoracoscopic lung biopsy and pleurodesis can also be a choice if the development of secondary spontaneous pneumothorax occurs.

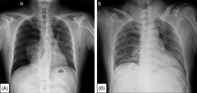

## INTRODUCTION

Pulmonary Langerhans cell histiocytosis (PLCH) is a rare disease caused by the proliferation of CD1a‐positive histiocyte‐like cells, which infiltrate the lung's interstitial layer. It was previously called eosinophilic granuloma of the lung, pulmonary Langerhans cell granulomatosis and pulmonary histiocytosis X. Most cases affect young to middle‐aged people, especially adult heavy cigarette smokers. PLCH is uncommon and its exact incidence and prevalence are unknown. In general, there is an equal distribution between males and females. Approximately, in Japan, the prevalence estimates are 0.27 per 100,000 in men and 0.07 per 100,000 among women. PLCH is estimated to account for 3%–5% of adult diffuse parenchymal lung disease. Surgical lung biopsy showed PLCH in less than 5% of cases. The characteristic of PLCH by the development of multiple cystic lung lesions and ground‐glass nodules may predispose to secondary spontaneous pneumothorax and atelectasis in 15%–25% of cases.

Medical imaging application for the diagnosis with high‐resolution computed tomography (HRCT) is effective in the follow‐up of PLCH because HRCT scan findings approximately reflect histopathological disease activity. The granulomatous process of PLCH affected small airways in an acinar distribution. Cystic lesions resulted from the destruction of the bronchiolar wall and progressive dilatation of the lumen, subsequently circumscribed by fibrous tissue, which may be demonstrated under the imaging examination follow‐up.

No effective treatment is available to date. However, the primary treatment for almost one third of the PLCH patient population is smoking cessation, and the empiric usage of corticosteroids and cytotoxic drugs is a standard practice. In many aspects, lung transplantation is a consideration—nevertheless, tobacco cessation therapy usually brings marked improvement evidenced by radiological examination. Early surgical intervention by minimal invasive thoracoscopic lung biopsy and pleurodesis can also be a choice if the development of secondary spontaneous pneumothorax occurs.

## CASE REPORT

Our patient was a 49‐year‐old male without any systemic disease, a heavy smoker of about 1–2 packs per day over approximately 30 years, without any specific or respiratory symptoms for the past years before presentation, without the need of taking any medication and without pulmonary hypertension on the record. He presented with non‐productive cough for about 3 months and progressing dyspnoea not related to the position changes but worsening with daily activity, respiratory distress and desaturation (smoking index [SI] = cigarettes smoked per day [CPD] × years of tobacco use. Grading of severity as mild ≤ 200, moderate 200–400 and heavy ≥ 400; the CPD estimated for current and former smokers).

**FIGURE 1:**
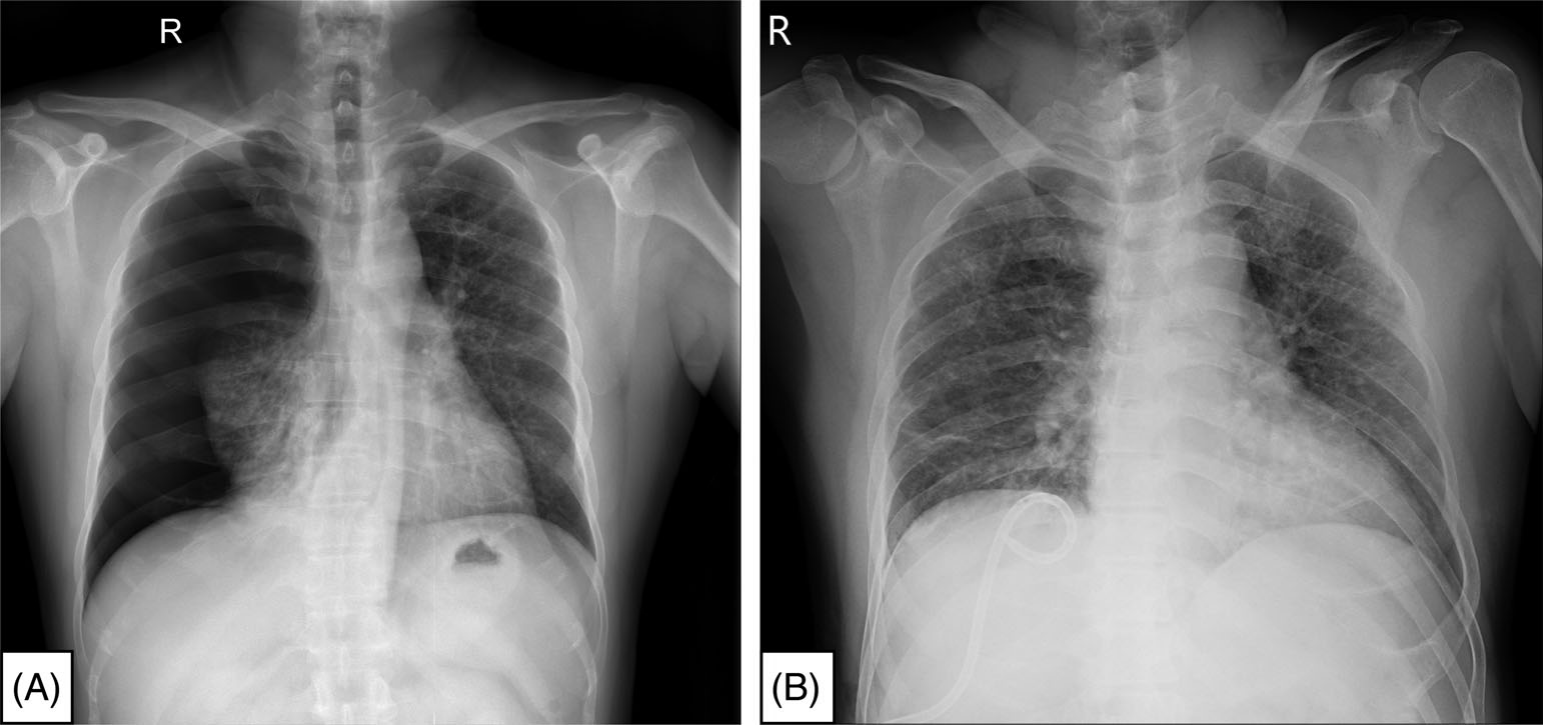
(A) Chest film showed right‐sided pneumothorax with total atelectasis. (B) Chest film showed right‐sided full lung expansion post‐pigtail chest drainage placement and bilateral multiple nodule opacities with cystic changes

Chest x‐ray (CXR) showed right‐sided pneumothorax with total atelectasis, with increased infiltration in the left lung (Figure 1A). Next, full lung expansion of the right lung post‐pigtail chest drainage was placed, followed by CXR, which demonstrated bilateral multiple nodule opacities with cystic changes (Figure 1B).

**FIGURE 2:**
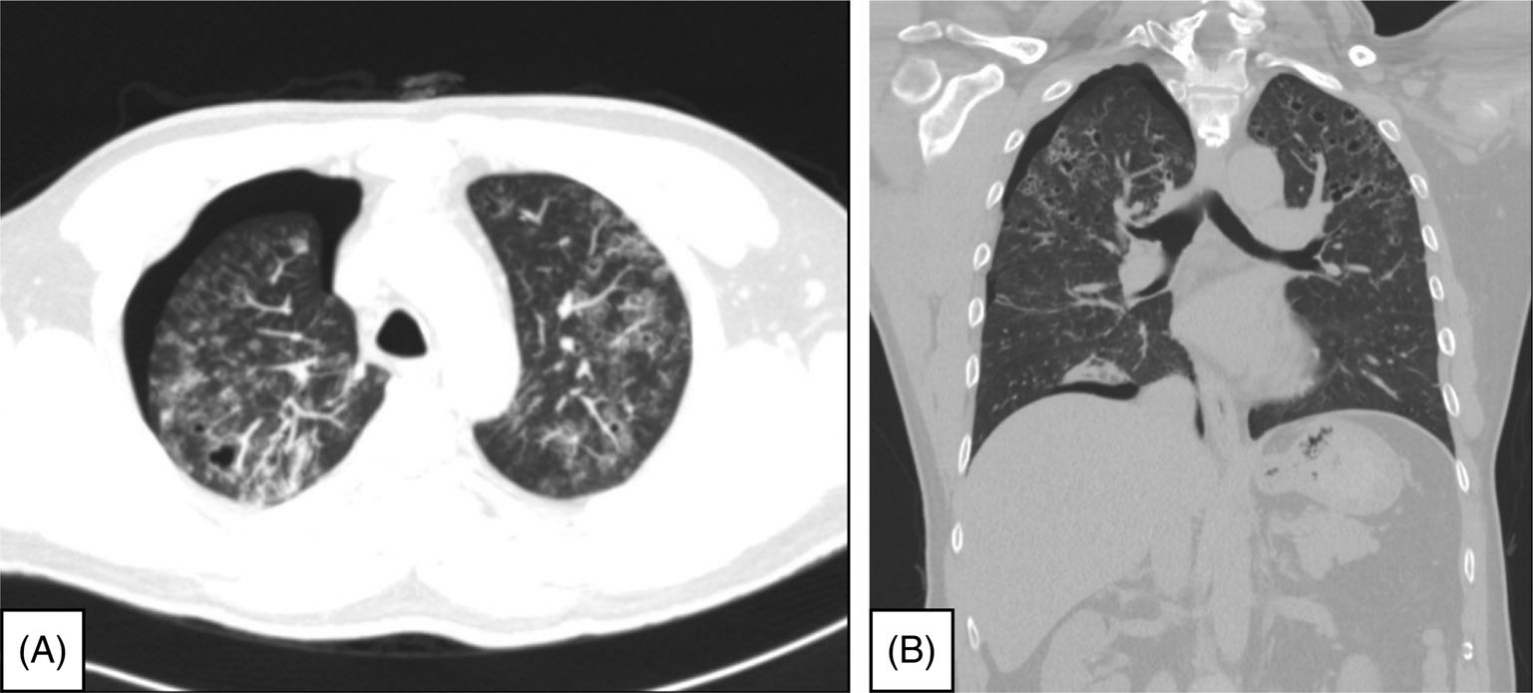
(A) Transverse view and (B) coronal view of the lung window of chest computed tomography. Right spontaneous pneumothorax and lung atelectasis. Bilateral multiple bizarre‐shaped, thick‐walled, ill‐defined, air‐density infiltrated parenchyma cysts; increased consolidations; and multiple ground‐glass cavitary nodules with interstitial thickening were noted

**FIGURE 3:**
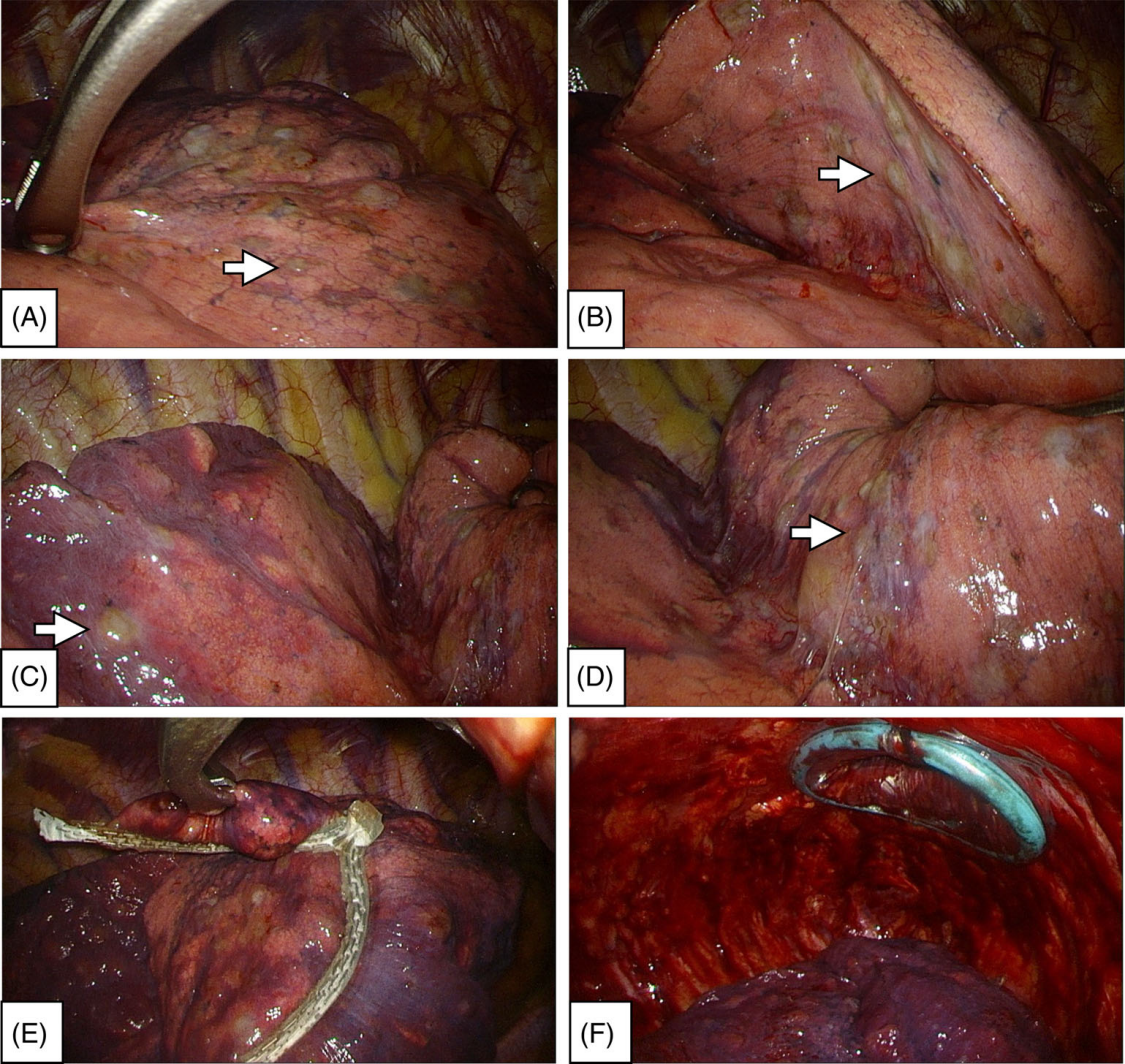
Intraoperative view under minimal invasive thoracoscopy approach. (A) Right upper lobe. (B) Fissure site of the right upper lobe. (C) Right lower lobe. (D) Right middle lobe. Multiple subpleural cystic fibrotic lesions (white arrows) over the entire right lung parenchyma. (E) Wedge resection of the right upper lobe of the lung by Endo GIA™ Reinforced Reload with Tri‐Staple™ Technology, Medtronic. (F) Pleurodesis

**FIGURE 4:**
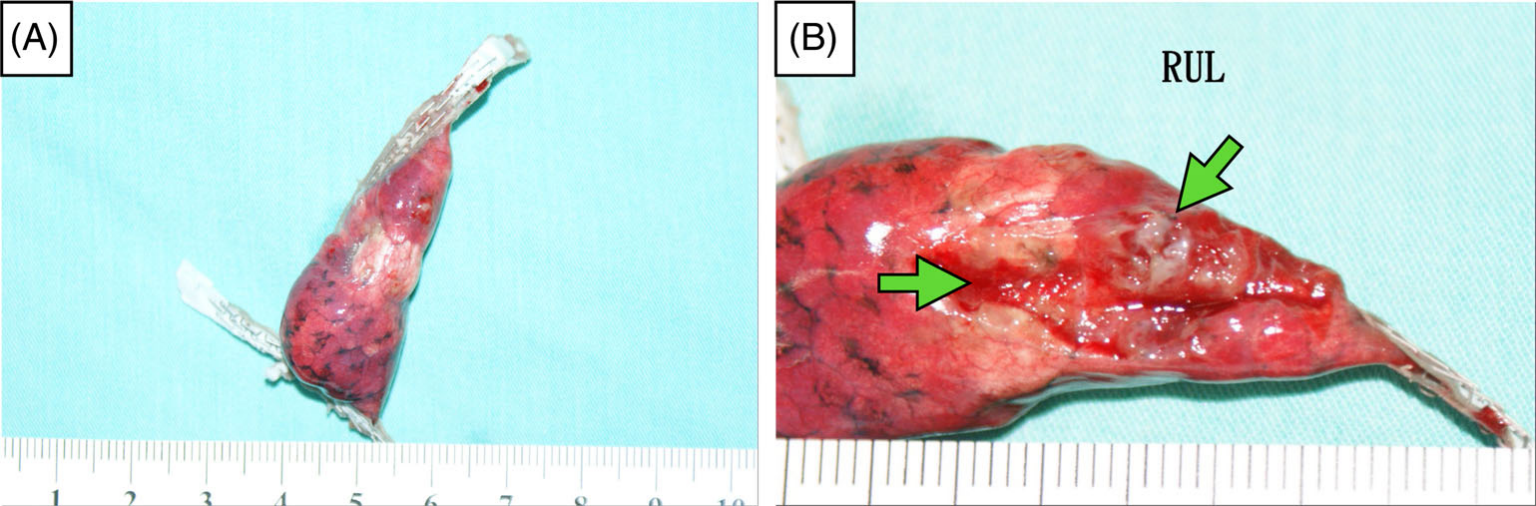
(A) Specimen of the right upper lobe of the lung with emphysematous changes and anthracosis lung. (B) Specimen of the right upper lobe of the lung with multiple subpleural cystic fibrotic lesions (green arrows)

Chest computed tomography (CT) revealed right‐sided spontaneous pneumothorax and lung atelectasis. Bilateral multiple bizarre‐shaped, thick‐walled, ill‐defined, air‐density infiltrated parenchyma cysts; increased consolidations; multiple ground‐glass nodules; and cavitary nodules with interstitial thickening wall throughout the entire lung were noted (Figure 2A, transverse view; Figure 2B, coronal view, lung window of chest CT). Due to persistent air leakage, surgical intervention with minimal invasive thoracoscopic lung biopsy with wedge resection of the right upper lobe of the lung and pleurodesis was done. During the operation, multiple subpleural cystic fibrotic lesions over the entire right lung parenchyma and persisted air leakage over the right upper lobe of the lung were noted (Figures 3A‐F, intraoperative view under minimal invasive thoracoscopy approach; Figure 4A,B, specimen of the right upper lobe of the lung). The post‐operative hospital course was uneventful.

**FIGURE 5:**
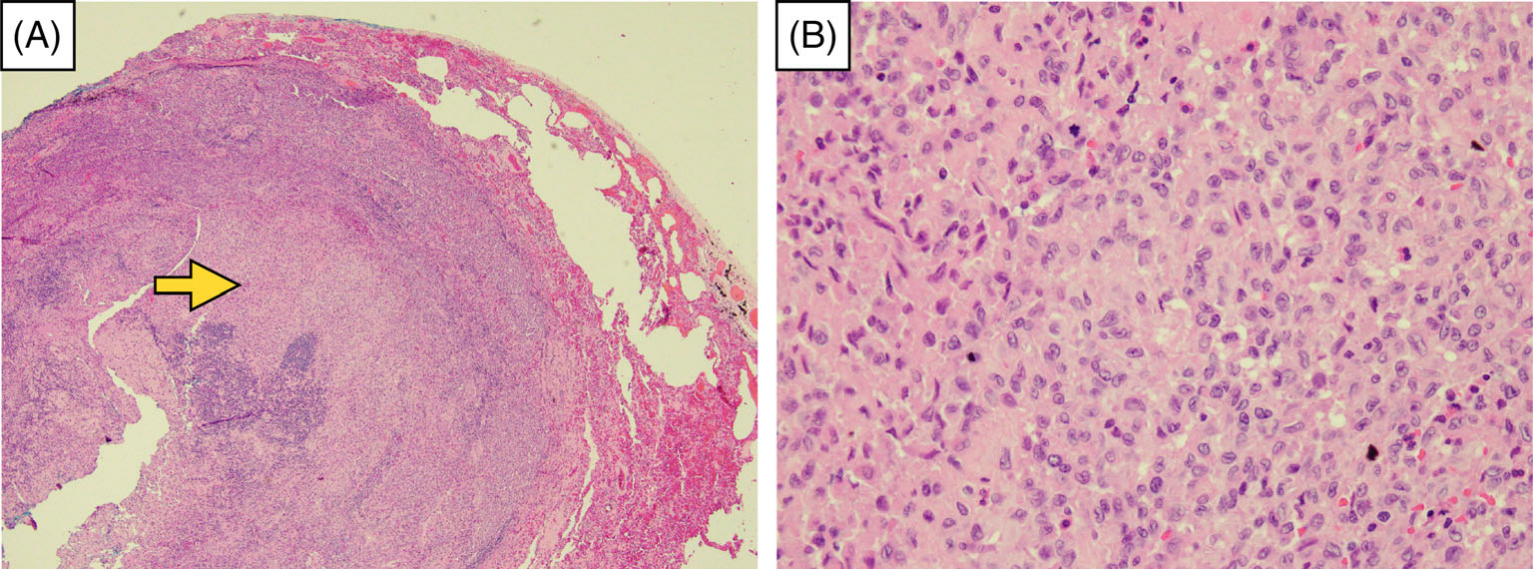
The specimen is under microscopic examination. (A) Histiocyte‐like cells aggregate with nuclear grooving in the pulmonary parenchyma (yellow arrow). (B) Higher magnification showing Langerhans cells with background inflammation cell infiltration including eosinophils, lymphocytes, plasma cells and neutrophils; characteristics of folded and convoluted

**FIGURE 6:**
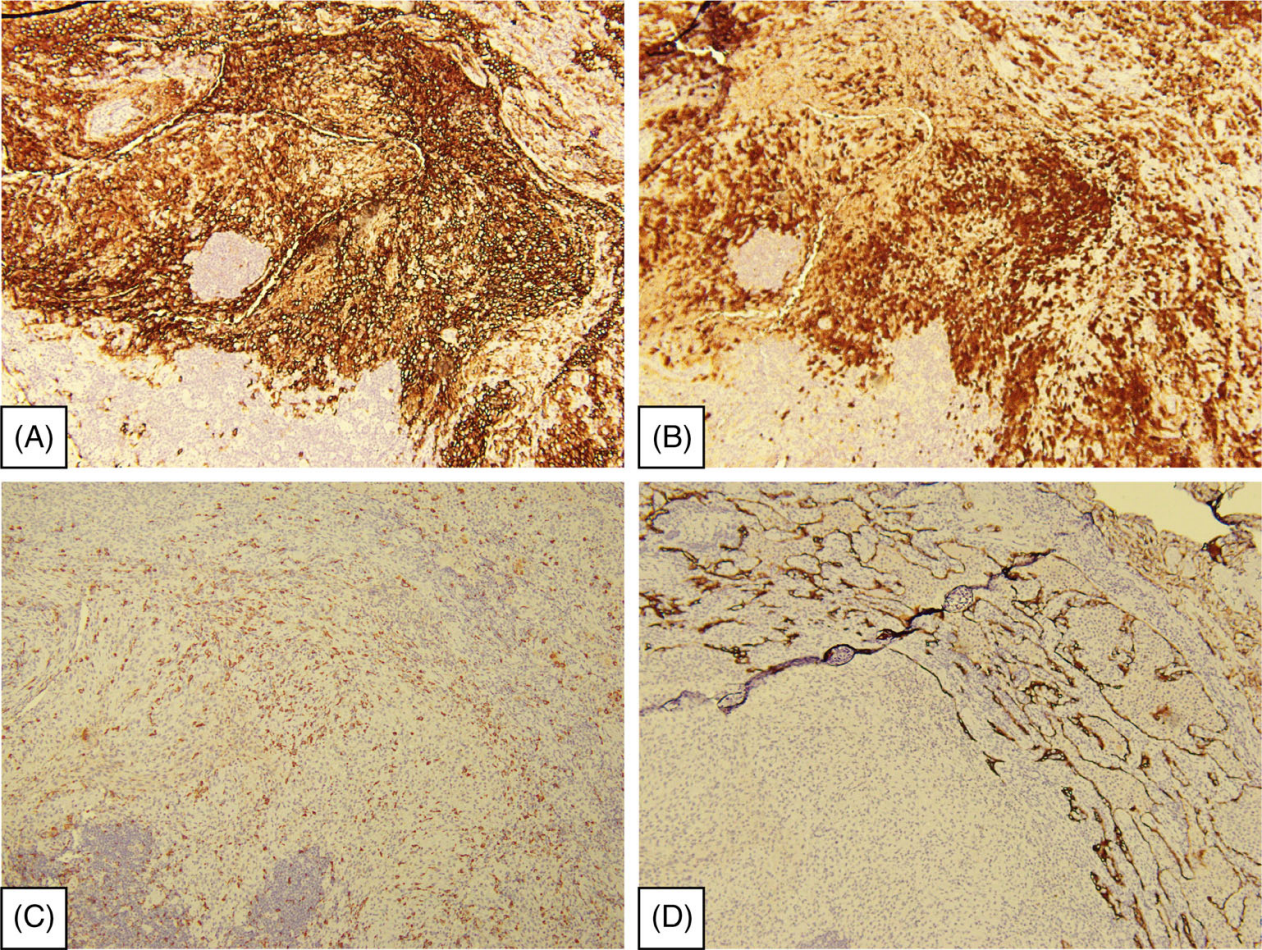
The specimen is under microscopic examination, and immunohistochemical stain. (A) CD1a (+). (B) S100 (+). (C) CD68 (+). (D) CK‐7 (−)

Pathology reported under the microscopic examination showed histiocyte‐like cell aggregates with nuclear grooving in the pulmonary parenchyma. Langerhans cells with background inflammation cell infiltration include eosinophils, lymphocytes, plasma cells and neutrophils, characteristics of folded and convoluted (Figure 5). Immunohistochemical stain demonstrates CD1a(+), S100(+) and CD68(+) variables. Acid‐fast stain, Periodic Acid‐Schiff (PAS) stain, CK‐7 stain and Grocott‐Gomori's methenamine silver (GMS) stain were negative (Figure 6). PLCH was diagnosed.

**FIGURE 7:**
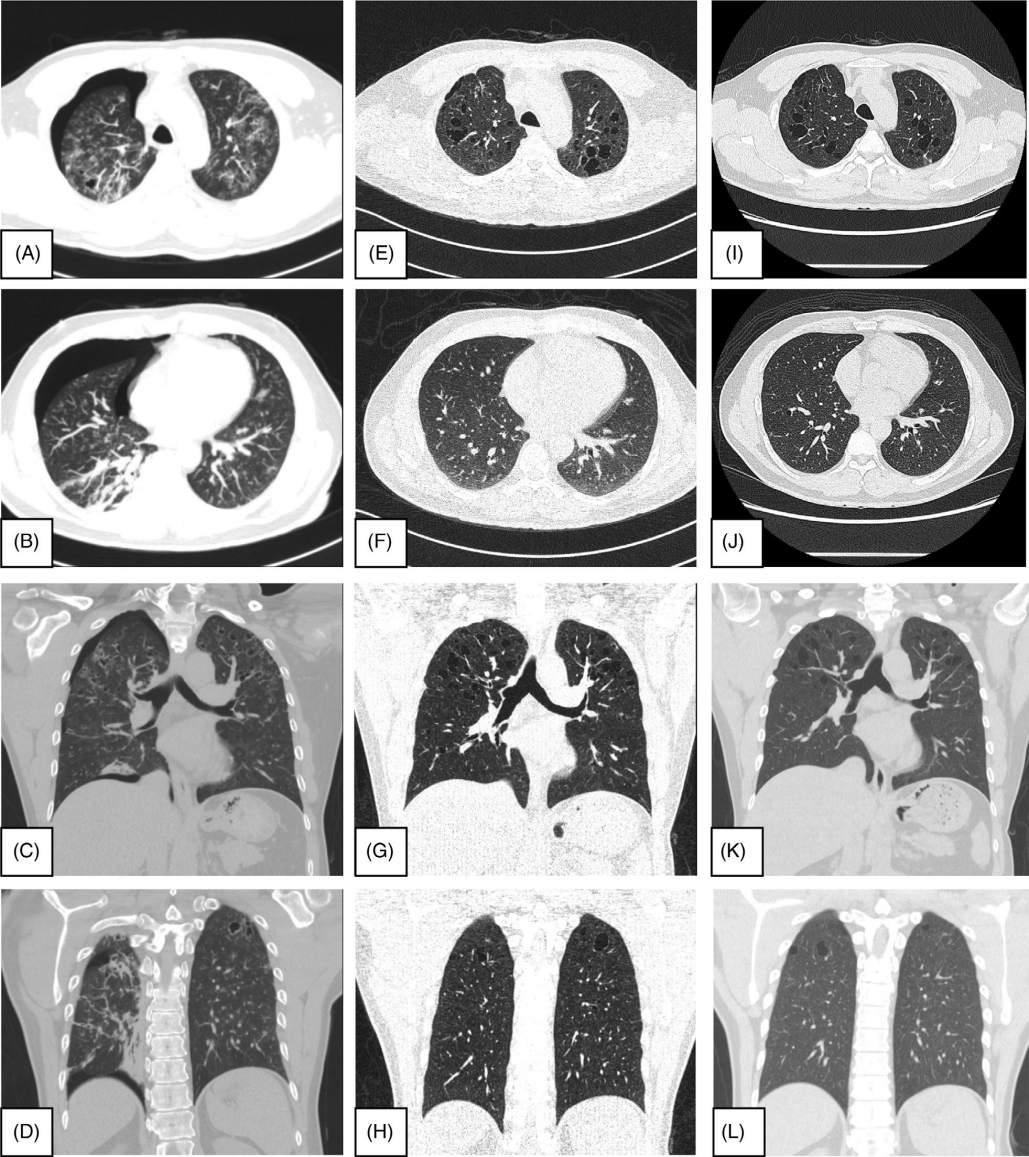
(A–D) Event initial episode. (E–H) Event after 3 months of smoking cessation. (I–L) Event after 14 months of tobacco cessation. Image examination by lung window of chest computed tomography via transverse and coronal view demonstrates bilateral lung, bizarre‐shaped and cavitary nodules, peripheral bronchioles tree in buds, various and numerous irregular air‐density parenchyma cysts, wall thickening improving without progression, decreased in size and numbers. Infiltration, consolidation, ground‐glass opacities and wall thickening of bronchioles became thinner and vanished

Smoking cessation was stated, and follow‐up conducted 3–14 months after smoking cessation showed, on bilateral lung chest CT, improvement and disappearance without progression of the bizarre shape and cavitary nodules, peripheral bronchioles tree in buds and various and numerous irregular air‐density parenchyma cysts. Wall thickening infiltrates cysts became thinner and decreased in size and numbers (Figure 7A–D, initial episode; Figure 7E–H, after 3 months of smoking cessation; Figure 7I–L, after 14 months of smoking cessation). There is no recurrent spontaneous pneumothorax at the outpatient department follow‐up after the invasive surgical procedures. During the imaging follow‐up, there was no extra‐specific empiric medication treatment needed, such as corticosteroids or any other cytotoxic drugs, because of his healthy clinical presentation record date until February 2022 (at the time of writing this case) since the event happened 2 years ago.

## DISCUSSION

After reviewing the literature, no effective treatment is available to date. The primary treatment of PLCH is smoking cessation, and the empiric usage of corticosteroids and cytotoxic drugs is a standard practice. In many aspects, lung transplantation may be needed considering haemodynamics and pulmonary–vascular circulation.

Kambouchner et al. report that HRCT is an effective imaging tool to follow‐up PLCH because HRCT scan findings reflect histopathological disease activity. The image evolution followed an orderly progression that encompassed part of the spectrum, from an early invasion of the bronchiolar wall by PLCH in three stages as florid cellular granuloma, in which bronchocentric cellular infiltrations of Langerhans cells form nodule. During the early fibrotic changes, Langerhans cells decrease in number, inflammatory cells become predominant and fibrotic changes begin. Furthermore, cicatricial fibrosis irreversibly destroys the lung structure as in end‐stage scarring status.^1^, ^2^

Kinoshita et al. report that lung transplantation and pulmonary arterial hypertension‐specific therapies have been recently reported to be effective in treating Pulmonary Langerhans Cell Histiocytosis ‐ pulmonary hypertension (PLCH‐PH), but its prognosis is still unfavourable. Possible aetiologies ascribed include the narrowing of pulmonary arteries due to the direct involvement of Langerhans cell granulomas, vascular remodelling of small pulmonary arteries in areas uninvolved in Langerhans cell granulomas and pulmonary vasoconstriction due to chronic hypoxaemia.^3^

Mogulkoc et al. reported that two patients with histologically proven PLCH underwent radiological improvement following smoking cessation. Bombesin‐like peptides (BLPs) are chemotactic for peripheral blood monocytes, stimulating the phagocytic function of tissue macrophages and promoting the release of cytokines such as interleukin‐1 beta and phagocytic, granulocyte, colony‐stimulating factors. It represented that many cigarette smokers have increased neuroendocrine cells and increased release of BLP levels. Therefore, such smokers may be at greater risk of developing smoking‐related lung diseases such as PLCH.^4^, ^5^, ^6^ Patients with PLCH require long‐term follow‐up to detect potential disease progression and relapse despite apparent clinical stability.

Mendez et al. reported that 16 of 102 patients (16%) with PLCH had pneumothorax, the mean age at the time of diagnosis was 29.4 years and all had smoked cigarettes. In addition, 63% had more than one episode. Recurrent pneumothorax percentage was as high as 58% to the ipsilateral side when it was managed only by observation or chest tube without pleurodesis and 0% after surgical management with pleurodesis.^7^ This result revealed the importance of the early use of surgical therapy with pleurodesis in managing patients with PLCH and spontaneous pneumothorax, which was proven successful throughout our case presented.

In conclusion, we report a successful case development of spontaneous pneumothorax with PLCH in an adult heavy cigarette smoker, after the early interventional surgical treatment by minimal invasive thoracoscopic lung biopsy and pleurodesis. After 3–14 months of follow‐up, there was no recurrence of spontaneous pneumothorax. The relationship between tobacco cessation behaviour and the practical improvement of radiological examination, without the need for any specific medication, substantially reduces the burden of side effects on the body associated with a long‐term reliance on steroid treatment, which provides a way of more remarkable improvement of respiratory impairment and healthy clinical presentation. Early surgical intervention management can also be an effective and practical therapy choice when there is development of secondary spontaneous pneumothorax.

## CONFLICT OF INTEREST

None declared.

## AUTHOR CONTRIBUTION

Chiao‐Yun Tsai wrote and edited the manuscript. Hsu‐Chih Huang, Jiun‐Yi Hsia and Chih‐Yi Chen reviewed the manuscript. Zhen‐Dong Xu provided the pathological images and illustrated advice. All authors participated in multidisciplinary team care.

## ETHICS STATEMENT

The authors declare that appropriate written informed consent was obtained for the publication of this manuscript and accompanying images.

## Data Availability

Data are available from the corresponding author upon reasonable request.
